# Co-Developing Skill Training with Older Volunteer Resource Navigators

**DOI:** 10.1093/geroni/igaf122.4368

**Published:** 2025-12-31

**Authors:** Patricia Oh, Elizabeth Gattine

**Affiliations:** University of Maine, Bangor, Maine, United States; State of Maine Governor’s Office, Augusta, Maine, United States

## Abstract

**Background:**

Older rural dwellers face significant barriers to essential resources, healthcare, and social connections. To increase access, the Governor’s Cabinet on Aging, UMaine Center on Aging, and five Area Agencies on Aging, launched the Community Connectors project, a volunteer neighbor-to-neighbor resource navigator model, in 12 age-friendly communities. Connectors were trusted community members with varying backgrounds and professional skills.

**Project:**

The research team and subject matter experts collaborated with the Community Connectors to co-develop skills training that supports age-friendly community volunteers and the resource navigation role.

**Method:**

Fourteen Connectors (age range 45 – 78) participated in this rapid realist evaluation. Semi-structured interviews (n = 14) and 2 focus groups with connectors and age-friendly leaders identified 15 skill chapter topics, structure, and components. Subject matter experts collaborated with Connectors to develop chapters tailored to adult learning styles, while plain language experts ensured accessibility for diverse educational backgrounds and non-native English speakers. Connectors reviewed skill chapters before distribution. Surveys (n = 67), semi-structured interviews (n = 23), and 2 focus groups (n = 13) collected data about the skill chapter experience.

**Results:**

During the initial 9 months, 156 age-friendly leaders and community volunteers completed skill chapters. Despite initial reluctance, 71% of Connectors (10/14) completed 10 skill chapters, earning Certificates of Age-Friendly Community Development. Themes identified included (1) microlearning; (2) applied reflection; (3) peer learning; and (4) connection to experts.

**Conclusion:**

This collaborative approach developed accessible training materials for older community volunteers serving as resource navigators. Replication may provide guidance to organizations training older adults for community-based roles in geographically diverse locales.

